# Globe thermometer free convection error potentials

**DOI:** 10.1038/s41598-020-59441-1

**Published:** 2020-02-14

**Authors:** Eric Teitelbaum, Kian Wee Chen, Forrest Meggers, Hongshan Guo, Nicholas Houchois, Jovan Pantelic, Adam Rysanek

**Affiliations:** 1 School of Architecture, Princeton University, Princeton, NJ USA; 2 Singapore-ETH Centre, ETH Zurich, Singapore, SIN Singapore; 3 Andlinger Center for Energy and the Environment, Princeton University, Princeton, NJ USA; 4 Center for the Built Environment, University of California, Berkeley, CA USA; 5 School of Architecture and Landscape Architecture, University of British Columbia, Vancouver, Canada

**Keywords:** Mechanical engineering, Characterization and analytical techniques

## Abstract

For thermal comfort research, globe thermometers have become the de facto tool for mean radiant temperature, *t*_*r*_, measurement. They provide a quick means to survey the radiant environment in a space with nearly a century of trials to reassure researchers. However, as more complexity is introduced to built environments, we must reassess the accuracy of globe measurements. In particular, corrections for globe readings taking wind into account rely on a forced convection heat transfer coefficient. In this study, we investigate potential errors introduced by buoyancy driven flow, or free convection, induced by radiant forcing of a black globe’s surface to a temperature different from the air. We discovered this error in an experimental radiant cooling system with high separation of air to radiant temperature. Empirical simulations and the data collected in a radiant cooling setup together demonstrate the influence of free convection on the instrument’s readings. Initial simulation and data show that *t*_*r*_ measurements neglecting free convection when calculating *t*_*r*_ from air temperatures of 2 K above *t*_*r*_ could introduce a mechanism for globe readings to incorrectly track air temperatures. The experimental data constructed to test this hypothesis showed the standard correction readings are 1.94 ± 0.90 °C higher than the ground truth readings for all measurements taken in the experiment. The proposed mixed convection correction is 0.51 ± 1.07 °C higher than the ground truth, and is most accurate at low air speeds, within 0.25 ± 0.60 °C. This implies a potential systematic error in millions of measurements over the past 30 years of thermal comfort research. Future work will be carried out to experimentally validate this framework in a controlled climate chamber environment, examining the tradeoffs between accuracy and precision with globe thermometer measurements.

## Introduction

We have discovered that millions of mean radiant temperature measurements made using globe thermometers could be systematically flawed, impacting thermal comfort research, building controls and modeling. Engineered solutions for providing thermal comfort in the built environment have historically prioritized one mode of heat transfer over all others: convection. While thermal comfort research for the human body has always considered all aspects of heat exchange including radiation and air speed, the majority of the systems being designed and analyzed in applied research tuned only temperature and humidity air properties for desired physiological responses^1–3^. Air became the primary medium of thermal intervention and the thermostat as its control mechanism. Local climate dictates air conditioner or furnace sizing based on the humidity and air temperature based loads. However, these systems fail to engage with radiation as an independent mechanism for comfort, despite equal radiative and convective fluxes in typical office environments^4^. This is largely due to the significant fundamental differences in the measurement and characterization of radiant heat transfer and convective heat transfer. Radiant heat transfer depends on surface temperatures whereas convection can be determined using simple air temperature measurements as proxies. It is not uncommon in the implementation of air-based systems to assume air temperature directly controls surface temperature and thus radiant exchange. But contemporary demands for improved efficiency and thermal comfort have increased interest in radiant heating and cooling systems and radiant impacts on comfort. This requires new analysis of radiant environments and the variation of the mean radiant temperature, the temperature defined by an imaginary uniform surface that imparts the same radiant exchange on a person as the space they are in. We propose a reevaluation of the way in which radiant exchange and mean radiant temperature are measured using globe thermometers in the built environment to improve contemporary building thermal comfort and efficiency analyses.

Building codes in countries or regions as climatically different as Singapore^5^, the European Union^6^, and the United States^7^ acknowledge the importance of both radiant and air temperatures, yet reinforce control practices that prioritize air measurements and avoid the challenges of radiant heat transfer evaluation. Radiant heat transfer, specifically the radiant heat exchange between human bodies and the environment surrounding them, has long-been observed to have a significant role in affecting thermal comfort^4^. However, the standard measurement technique for radiant heat transfer in the built environment is a globe thermometer, and is susceptible to many issues, such as convection^8–10^, sensor position^2^, and coating^11^.

To quantitatively characterize the radiant environment in a building, blackened globe thermometers are often used to measure a “globe temperature” which is used to approximate the mean radiant temperature, *t*_*r*_. Mean radiant temperature is a simplification for radiant heat transfer, defined as the uniform temperature of an imaginary enclosure in which the radiant heat transfer from the human body is equal to the radiant heat transfer in the actual non-uniform enclosure^12^. Globe thermometers operate on an assumed principle that a thin, near mass-less blackbody sphere will interact radiatively with all surfaces surrounding it and arrive at an equilibrium temperature near or equal to the mean radiant temperature of its surrounding environment. This type of measurement technique rose to prominence in 1934 when a method of correcting measurements for wind speed was published^9^, and has remained unchanged. Several characterizations of the heat transfer properties characteristics of the globes have been conducted^13,14^, and a widely accepted correction factor for forced air movement was proposed in 1987 by Richard de Dear^8^, shown in Eqs. 1 and (2). In these equations, *t*_*r*_ is the mean radiant temperature [*K*], *t*_*g*_ is the measured globe temperature [*K*], *ϵ* is the emissivity of the globe [0.95], *D* is the diameter of the globe [*m*], *t*_*a*_ is the air temperature [*K*], *h*_*c*_ is the forced convective heat transfer coefficient [*Wm*^−2^ *K*^−1^], and *v*_*a*_ is the air speed [*ms*^−1^].

1 $${t}_{r}=\sqrt[4]{{t}_{g}^{4}+\frac{{h}_{c}}{\varepsilon \cdot {D}^{0.4}}\cdot ({t}_{g}-{t}_{a})}$$

2 $${h}_{c}=1.1\cdot 1{0}^{8}\cdot {v}_{a}^{0.6}$$

Researchers have noted the limitations of black globes in outdoor environments and windy environments^10,15,16^, noting the tradeoffs between size and sensitivity to convection and radiation. Thorsson *et al*. derived a subsequent correction factor for use in direct solar radiation^10^. Importantly, it has been noted that black globes often track air temperature, a phenomenon that can be mitigated in the presence of detectable air motion thanks to the forced convection correction factor in Eq. 1. Additionally, other researchers have previously noted potential sensitivities of black globe readings to the emissivity of their often visibly black coating, along with an inability to resolve spatial or directional variations in the readings and a high sensitivity to the air velocity measurements that can turn the device into more of an anemometer than a radiant temperature sensor^11^.

In an analysis by Humphreys in 1977^17^, there is an experiment conducted for free convection with a subsequent analysis demonstrating that for a large *t*_*r*_ to *t*_*a*_ difference, the resulting *t*_*g*_ for globe thermometers of different diameters would be decrease as globe diameter increases. It is implied that convection is the driving force for this relationship, but does not use a free convection heat transfer coefficient despite low air speeds of 0.1 *ms*^−1^ in experiments. Instead, it is assumed that radiant and convective heat transfer are similar in magnitude, citing a radiant response ratio *H*_*r*_ of 0.5 to account for this equivalence which is propagated through the analysis, arriving at a maximum potential error of 0.6 °C for *t*_*r*_ − *t*_*a*_ of 5 °C. Radiant response ratio is defined in Eq. 3, and the proposed term for *h*_*r*_ in Eq. 4. In Eq. 4, *σ* is the Stefan-Boltzmann constant, 5.67 ⋅ 10^−8^  *Wm*^−2^  *K*^−4^.

3 $${H}_{r}=\frac{{h}_{r}}{{h}_{c}+{h}_{r}}$$

4 $${h}_{r}=4\varepsilon \sigma {t}_{r}^{3}$$

The simplification of Eq. 3 to 0.5 occurs in spite of a chart for generalizing the calculation of *H*_*r*_, and relying on a probabilistic model which states that the radiant response ratio falls between 0.42 and 0.5 with 95% probability. The same assumption was used by Fountain in 1987^13^. The combination of these oversights fails to address how small ping pong balls as Fountain studied, or any sized globe, would respond to buoyancy driven flow imparted by large *t*_*r*_ to *t*_*a*_ differences resulting in free convection. The experimental section was conducted only for radiant heating, and with a Nusselt number correlation that is now outdated. Similar research examining extreme radiant heating conditions with globe thermometers was conducted by Graves in 1974, where extreme room surfaces above 100 °C^18^ generated high globe temperatures and detectable buoyancy flow around globes, yet less extreme conditions were not considered, and thus the analysis failed to characterize typical radiant system regimes where free convection could still manifest around globes at lower temperatures.

Perhaps most importantly, these analyses fail to account for nonlinear changes in convective heat transfer associated with changing air movement. Therefore the cumulative heat loss due to convection must account for nonlinear effects across nonuniform air speeds. The kata thermometer was an early device used to account for nonlinear differences and variation of convection along with radiant exchanges, which used both a silvered sphere to minimize radiant heat exchange and isolate convection and a blackened sphere both maintained at constant temperature while recording the watts of heat inputs into the globes for comparison^19–21^. These studies cause much internal discussion among the American Society of Heating and Ventilating Engineers regarding the relationship of radiation and thermal comfort^22,23^. It is not until 1934 that Bedford and Warner use the kata thermometer for its high sensitivity to air movement as a correction for globe thermometer radiant experimentation^9^. Bedford’s paper which has become a seminal piece in the field, states that at low air velocities, the convective exchange increases more rapidly than *t*_*g*_ − *t*_*a*_ increases, implying that the convective heat transfer coefficient must be modified for free convection, “but for the purpose of this paper it did not appear to be necessary to derive an equation of more complex form...”. The presence of free convection did not go unnoticed from subsequent researchers^13^, however the contribution was not further investigated since many real building scenarios do not generate large air temperature to surface temperature separations. Yet with new advancements with radiant cooling systems which separate radiative from convective heat transfer, it becomes a valid question to test the limits of black globes, with respect to greater air/surface separations for radiant comfort systems.

Recent work by the authors in evaluating an outdoor radiant cooling pavilion has provided field-based evidence elucidating the true magnitude of free convection contributions that have been mentioned in the literature, but have yet to be directly addressed. In the pavilion, we maintained conditions with *t*_*r*_ up to 6 °C below *t*_*a*_. In the absence of air motion, Eq. 1 shows *t*_*r*_ to be equal to *t*_*g*_, neglecting the potential for free convection that the radiant environment could generate around the sphere. Comparing the mean radiant temperature calculated from globe readings with the ground-truth mean radiant temperature established with a pyrgeometer and pyranometer, it was immediately clear that the application of the standard correction factor using measured wind speed to derive the mean radiant temperature did not produce an accurate *t*_*r*_, particularly for low wind speeds, indicating that another heat exchange mechanism is occurring independent of the wind speed, which we hypothesized was free convection. While free convection is accounted for in ISO 7726^12^ and examples of the ISO 7726 correction framework exist in research^24^, we present a methodology to unify both free and forced with a mixed convection approach.

We measured data in the pavilion with globes that did not appear to reflect the environmental conditions that the researchers physiologically experienced. We outfitted the space with more accurate sensing equipment and confirmed that the globes did not produce high fidelity readings in many operating conditions. To understand why, we formulated a convective and radiative energy balance model around a globe thermometer from empirically derived heat transfer equations to describe the potential contribution to globe measurement errors from free convection, presented here in a generalizable framework. In the literature it has been mentioned that “natural [free] convection currents affect heat exchange, and modify the relations”^14^, however the precise corrections have never been directly addressed for a number of reasons. For instance, in Hey’s analysis^14^ from 1968, the goodness of fit of several different convective heat transfer coefficients is demonstrated, all for forced convection. The primary contribution of this paper is data demonstrating a need for a generalizable approach to convective corrections, also showing how a generalizable Nusselt number correlation framework through which forced, free, and mixed (both free and forced) convection’s effects can be used to determine a globe thermometer’s *t*_*r*_ reading.

## Results

### Simulation results

Comparing the contributions from both free and forced convection to the globe temperature, as the air velocity increases, forced convection will dominate the reading for *t*_*g*_. However, as the difference between *t*_*g*_ and *t*_*a*_ grows, there is a plateau in the free convection response. The magnitude is smaller than air speeds above 0.1 m/s, however the plateau shape of the free convection response presents certain challenges for interpreting the true *t*_*r*_ from globe readings in a free convection dominated scenario. For example, the change in *h*_*c*_ for *t*_*g*_ − *t*_*a*_ from 3 to 4 is approximately 8 to 8.2 *Wm*^−2^ *K*^−1^, therefore making the calculation very susceptible to any fluctuations. This is unlike the globe’s response to forced convection, which is nearly a linear response to air speed.

**Figure 1:**
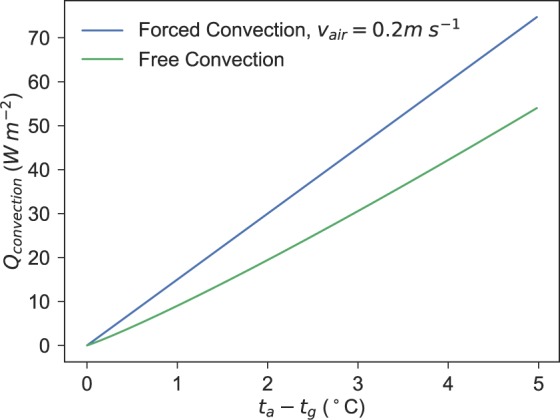
Comparison between heat lost due to free and forced convection.

However, as *h*_*c*_ is also multiplied by the temperature difference, the effect is not negligible. As shown in Fig. 1, the heat lost due to forced and free convection, *Q*_*convection*_ at a low air velocity is larger than the heat lost due to free convection, however the free convection portion is certainly not negligible.

**Figure 2:**
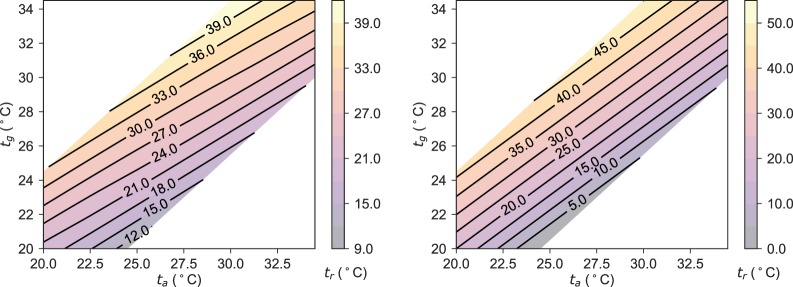
(a - left) The remapped domain calculating *t*_*r*_ from *t*_*g*_ and *t*_*a*_ measurements, correcting for free convection. (b-right) The remapped domain calculating *t*_*r*_ from *t*_*g*_ and *t*_*a*_ measurements, correcting for mixed convection for a fixed air speed of 0.3 *ms*^−1^.

Reconstructing a potential measurement domain with the free convection correction is shown in Fig. 2a, for differences in *t*_*a*_ and *t*_*g*_ up to 5 *K*. Fig. 2b shows a remapping of the domains for mixed convection with a fixed air speed of 0.3 *ms*^−1^.

This analysis illustrates the significance of free convection relative to forced convection, implying that free and mixed mode mechanisms may skew *t*_*r*_ calculations for larger *t*_*a*_ − *t*_*r*_ separations. The mixed corrections are quite significant, demonstrating how powerful forced convection is relative to free convection.

### Experimental results

**Figure 3:**
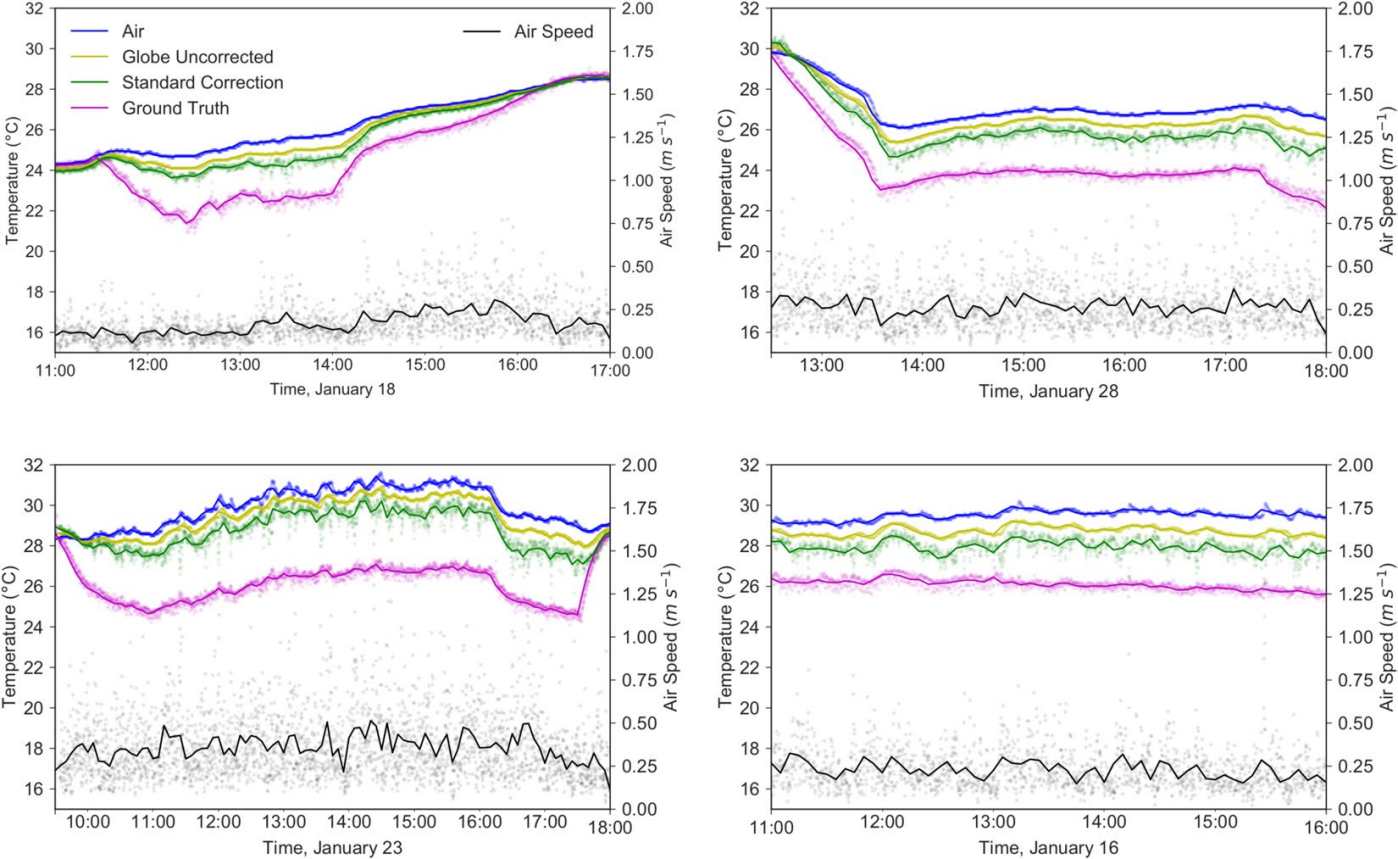
Datasets showing potential error using black globes to measure *t*_*r*_. From left to right, top to bottom, datasets were taken on January 18, 28, 23, and 16 of 2019. 10 second time averaged data is shown with translucent dots, and the 5 minute time average is shown with a solid line. Air speed has the largest variability.

As expected from the analysis in the previous section, data for *t*_*r*_ collected in the Cold Tube with globe thermometers was inconsistent with the ground truth *t*_*r*_ readings from the pyrgeometers. Figure 3 shows representative datasets of discrepancies between both methods.

 Figure 3 show that although the *t*_*r*_ readings from globes in this environment often follow the same form as the *t*_*r*_ ground truth measurements from the pyrgeometer, the globe’s reading does not deviate from *t*_*a*_ nearly as much as the pyrgeometer’s readings. The data shows the system turning on, turning off, with data diverging within the first 30 minutes of “on” operation.

**Figure 4:**
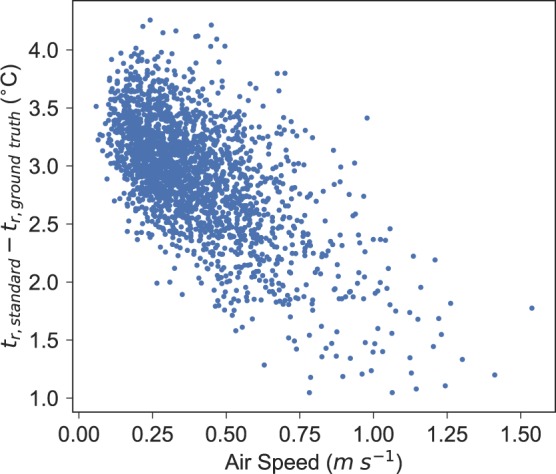
The difference in *t*_*r*_ measured by the globe thermometer (standard) and pyrgeometers (ground truth), plotted against air speed.

Further analysis examined the difference in the standard method of calculating *t*_*r*_ and the ground truth method using the pyrgeometers, plotted against air speed, to understand the behavior further. This analysis is shown in Fig. 4.

There is a slight downwards trend to the data (*r* = 0.62), implying that as the air speed increases, there is a tendency towards fidelity with the original correction from Eq. 1. The difference of the data with low air speeds indicates that the globe is highly sensitive to convection, an unavoidable phenomenon particularly in outdoor environments.

The turbulence intensity of this data was 0.42  ± 0.12, computed over 100 second rolling windows. There is a very slight negative trend between the difference between the two *t*_*r*_ methods and turbulence intensity (*r* = 0.22). Because of this turbulence intensity, most data was not collected under steady state conditions, thus the importance of a 5 minute time average. However, findings demonstrate that the mixed convection correction may not be sensitive to non-steady state conditions.

**Figure 5:**
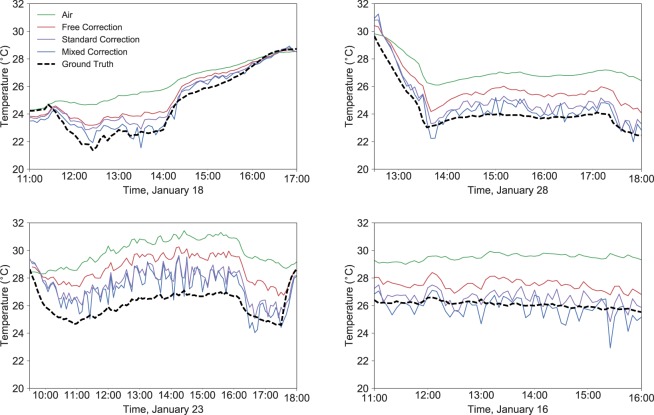
Datasets showing both free convection and mixed convection corrections for the data shown in Fig. 3, using *n* = 4 for the mixed convection *Nu* calculation at a 5 minute time average (Eq. 12). From left to right, top to bottom, datasets were taken on January 18, 28, 23, and 16 of 2019.

 Figure 5 shows free convection and mixed convection (*n* = 4 from Eq. (12)) corrections for the data presented in 3. Often the free convection correction still largely underestimates *t*_*r*_, since there is correspondingly little separation between *t*_*g*_ and *t*_*r*_. The mixed convection correction is frequently significantly closer to the ground truth *t*_*r*_ value, however the correction is quite noisy since there is an enhanced sensitivity to air speed.

**Figure 6:**
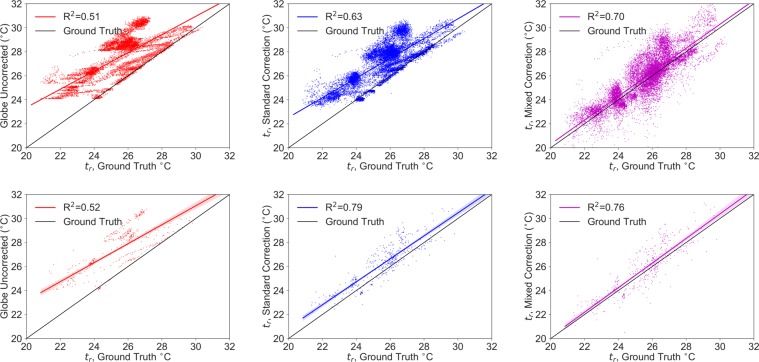
Correlations of *t*_*g*_, *t*_*r*_ from the standard correction, *t*_*r*_ with a mixed convection correction are compared to the ground truth for all data at a 10 second time average (top) and a 5 minute time average interval (bottom).

The magnitude of this deviation is illustrated in Fig. 6 where correlations for *t*_*g*_, *t*_*r*_ from the standard correlation, *t*_*r*_ with a mixed convection correction are compared to the ground truth. This plot serves to demonstrate both the noise associated with convection and the degree to which the existing approximation overestimates *t*_*r*_. For all of the data, the standard correction readings are 1.94 ± 0.90 °C higher than the ground truth readings for all measurements taken in the experiment. The proposed mixed convection correction is 0.51 ± 1.07 °C higher than the ground truth, despite the non-ideal laboratory conditions during testing. While the standard correction approximates *t*_*r*_ well for a 5 minute time average, the new proposed mixed correction framework is not sensitive to the smoothing interval.

## Discussion

**Figure 7:**
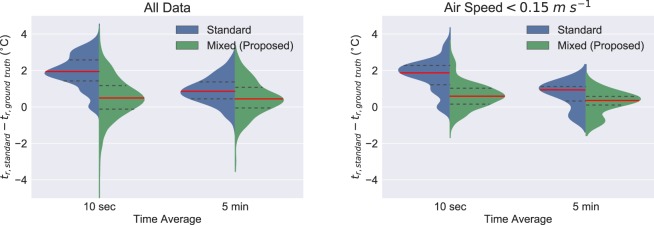
(a - left) Comparison of the standard and mixed convection corrections for all experimental data during experiments at 10 second and 5 minute time averages and (b - right) data at low air speeds of less than 0.15 *ms*^−1^. Red is the mean value, and the shape is the data distribution.

The results are analyzed further in Fig. 7a and b, examining the data distributions for both the 5 minute time average in addition to the 10 second time average. Two separate cases were examined, with 7a containing all of the data during system operation as shown in Figs. 5 and with 7b containing all of the data for low air speeds, *v*_*air*_ < 0.15  *ms*^−1^. As expected, the distributions of all mixed convection ranges never have a mean greater than 0.5 °C different than the ground truth and are significantly less sensitive to the long 5 minute time average. We hypothesize this is because the mixed convection coefficient corrects small time scale free convection phenomena, rather than just longer time scale forced convection providing a more realistic representation of the physical sensor. For low air flows, the distribution is narrower for the mixed convection case, also as expected since the standard correction cannot properly describe these low flow phenomena. These distributions are summarized in Table 1. When accounting for mixed convection, low air flow speeds are more accurately converted to *t*_*r*_ readings than with the standard correction.

**Table 1:** Mean, median, and standard deviation for each *t*_*r*_ − *t*_*r*,*groundtruth*_ measurement shown in Fig. 7. All values are in °C.

10 sec	Mean	Median	St. Dev.	5 min	Mean	Median	St. Dev
Standard	1.94	1.95	0.90	Standard	0.91	0.86	0.74
Mixed	0.51	0.49	1.07	Mixed	0.49	0.44	0.91
*v*_*a*_*ir* < 0.15	**Mean**	**Median**	**St. Dev**.	*v*_*a*_*ir* < 0.15	**Mean**	**Median**	**St. Dev**
Standard	1.72	1.87	0.86	Standard	0.67	0.94	0.70
Mixed	0.63	0.60	0.72	Mixed	0.25	0.35	0.60
*t*_*a*_ − *t*_*r*_ > 4	**Mean**	**Median**	**St. Dev**.	*t*_*a*_ − *t*_*r*_ > 4	**Mean**	**Median**	**St. Dev**
Standard	3.11	3.13	0.4	Standard	1.36	1.29	0.56
Mixed	1.63	1.71	0.90	Mixed	0.89	0.79	0.61

**Figure 8:**
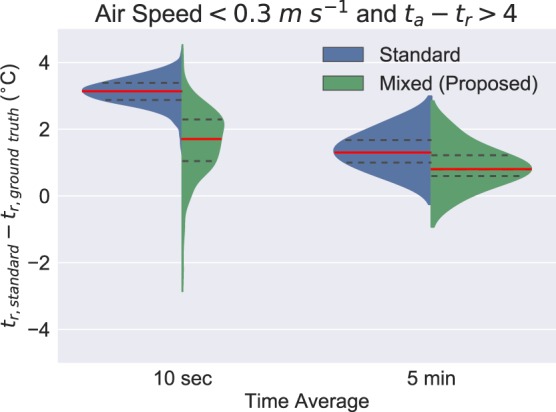
Data from less than 0.3 *ms*^−1^ air speed and greater than 4 °C *t*_*a*_ − *t*_*r*_ difference when using the *t*_*r*_ ground truth measurement. Red is the mean value, and the shape is the data distribution.

The mixed correction factor always outperforms the standard correction method. However as the radiative forcing of the globe thermometer reading increases to *t*_*a*_ − *t*_*r*_ > 4 at low air speeds of less than 0.3 *ms*^−1^, the standard correction is 1.63 ± 0.90 °C above the ground truth, compared to the mixed convection correction, which is only 0.89 ± 0.61 °C above the ground truth reading. This data is visualized in Fig. 8. At fast time averages, neither factor performs well, but even at 5 minute averages Fig. 8 demonstrates that small perturbations skew the reading as calculated with the standard correction.

Data presented herein demonstrate a significant contribution to globe temperature from free convection in environments with increasing air/surface gradients. The magnitude of this may affect sensor measurements and therefore comfort characterization. Similarly, steady state conditions may be difficult to ensure in the built environment outside of laboratory conditions, so being able to account for these nonidealities within an empirically derived framework could prove useful for thermal comfort researchers.

Predicted Percentage of Dissatisfied (PPD) was chosen as an example metric for comparing the impact of using the standard correction versus the ground truth values for *t*_*r*_, as the PPD framework uses *t*_*r*_ as a primary input, along with *t*_*a*_, *v*_*a*_, %RH, clothing insulation level (clo), and metabolic rate, (met). A metabolic rate of 1.2 met and clothing level of 0.5 clo were used as fixed inputs. PPD has a tangible output easily understood by a general audience with minimal context, describing the percent of the population that is predicted to be dissatisfied with their thermal environment based on an exponential probability density function derived by comparing the amount of heat in *Wm*^−2^ an individual is able to shed by convection and radiation at steady state in the measured environment compared to the person’s metabolic rate.

**Figure 9:**
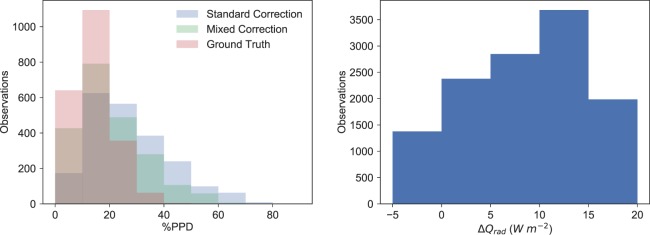
(a - left) Change in %PPD for the environmental conditions measured with the standard correction, mixed correction, and ground truth. (b - right) The difference *ΔQ*_*rad*_ in *Wm*^−2^ an individual would exchange with an environment measured to have *t*_*r*_ with a globe thermometer corrected with the standard method compared to the ground truth *t*_*r*_.

Examining the data presented here within the PPD framework, changing the measurement tool alone would significantly shift predicted occupant perception of the Cold Tube. Shown in Fig. 9a is a histogram of %PPD calculated from the Cold Tube environmental parameters collected on January 16th. The histogram contains *t*_*r*_ measured by the globe thermometer with the standard correction, the mixed convection correction shown in Fig. 5, and the ground truth. This data is also presented as the difference in radiant heat transfer potential between the standard convection correction and ground truth, shown in Fig. 9b. In this histogram, the net difference as calculated with Eq. 17 is shown. For the ground truth data, the PPD distribution is narrow. The difference in the *Wm*^−2^ distribution in Fig. 9 is large, and is indicative of substantially different comfort perceptions. The mean, median, and standard deviation for all data in Fig. 9a,b is shown in Table 2.

**Table 2:** Mean, median, and mode for each *t*_*r*_ measurement informing the %PPD calculation.

	Mean	Median	St. Dev.
Standard Correction	27.3	24.9	14.3
Mixed Correction	20.9	17.9	12.3
Ground Truth	14.4	13.03	6.8
*ΔQ*_*rad*_ (*Wm*^−2^)	9.1	9.8	6.5

Despite the mixed convection correction reducing the PPD to 20%, the distribution is quite wide and is still far from the value calculated with the ground truth. Providing better, higher fidelity *t*_*r*_ measurements may help thermal comfort prediction accuracy and overall comfort characterization. The difference *ΔQ*_*rad*_ in *Wm*^−2^ that an individual would radiantly exchange in a space with *t*_*r*_ measured with the standard correction from a globe thermometer compared to the ground truth established with the pyrgeometer cube is shown in Fig. 9b.

Despite the unique environment created in the Cold Tube, many real buildings can have significant *t*_*r*_ variation that may have been mischaracterized by standard globe corrections. The original globe studies by Bedford and Warner in 1934^9^ examined several scenarios with large *t*_*r*_ gradients in factories. Environments with exterior windows and walls and low air speeds could have also been mischaracterized as demonstrated by the data in this paper.

**Figure 10:**
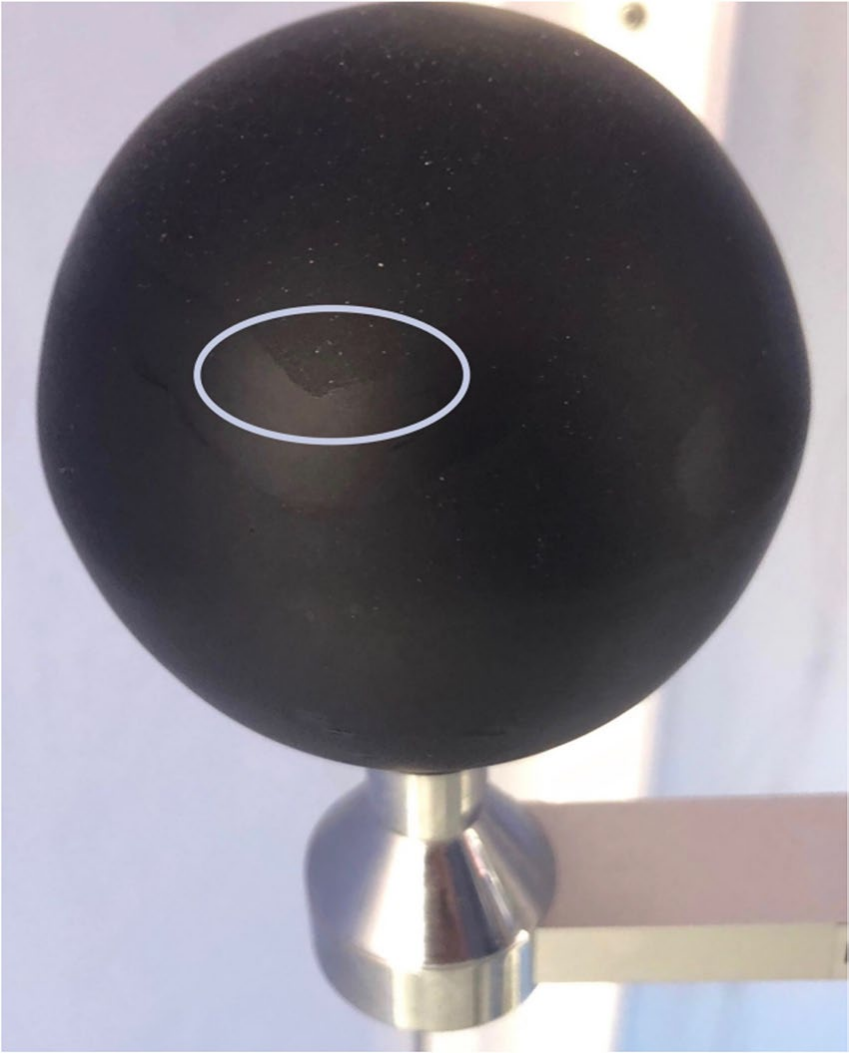
Condensation on a globe thermometer, with a cleaned region circled, removing condensation locally.

Unique to the Cold Tube was the demonstration that a globe thermometer could be radiatively forced to condense moisture, shown in Fig. 10. This condition only occurred when icewater was supplied as the cooling fluid, significantly lower than the typical 13–17 °C supply temperatures used during the course of experiments shown in this paper. Condensation on the globe indicates the heat of condensation is added to the energy balance, providing an additional source of error in certain environments, but more importantly exposes another mechanism that proves globes are not suitable for certain niche applications.

More broadly, the standard correction has been the predominant method used for over 30 years to correct globe thermometer readings^12^. These measurements have been used in thousands of thermal comfort studies, including 81,846 complete sets of objective indoor climate observations in the ASHRAE Global Thermal Comfort Database II^25^. These datapoints all use globe thermometers to measure *t*_*r*_ with the existing standard correction for forced, but not free and subsequently not mixed, convection. The analysis and data demonstrated in this paper shows that not only could the values be wrong for *t*_*r*_, but the differences could affect conclusions drawn from this database regarding thermal comfort.

In practice, these findings have far-reaching implications for thermal comfort and thermal comfort research, both in buildings with radiant systems and all-air comfort systems, which are much more prevalent. Simulations showing a discrepancy between the measured and simulated mean radiant temperatures^26^ in buildings with all air systems could be attributed to free convection contributions to black globe measurements. This systematic measurement error could explain such discrepancies. Additionally, studies seeking to understand thermal perception at the boundaries of comfort zones^27^ may be most at risk for misattributing causes of discomfort without adequately extracting mean radiant temperatures from globe readings. Further, using air temperature setbacks for energy savings^28^ in buildings will rely on better estimations of the mean radiant temperature^29^.

The data presented in this paper is a first step at demonstrating the significance of free convection effects around globes. The “Cold Tube” experiment designed in Singapore was not directly meant to test this effect, but the data demonstrated an alarming entanglement with free convection. Future work under controlled indoor laboratory conditions will be performed to further describe this effect, with higher confidence intervals for the correction shown in the discussion.

### Conclusions

The simulations and datasets presented in this paper make a compelling argument for the necessity of a mixed convection correction as the gradient between the air and surface temperatures in a space increases. Free convection effects can introduce a potential measurement error that is not currently accounted for, and is of the same magnitude as forced convection. This paper presents a simulation-based framework for free and mixed free with forced convection around a globe, and subsequent empirically derived correction factors to remap the measurement domain. The results demonstrate that free convection can play a significant role in skewing globe thermometer readings. Since this model was built up from empirical simulations, the new framework appears to be more robust for capturing smaller timescale convection perturbations from steady state. Data from an experimental setup in Singapore confirms this, showing a discontinuity between corrected globe temperature readings and ground truth mean radiant temperature readings. Specifically, the standard correction readings are 1.94 ± 0.90 °C higher than the ground truth readings for all measurements taken in the experiment. The proposed mixed convection correction is 0.51 ± 1.07 °C higher than the ground truth, and is most accurate at low air speeds, within 0.25 ± 0.60 °C. Using the mixed correction correction, *t*_*r*_ measurements from globe readings respond much more quickly to radiant and convective forcing, which could be useful for comfort characterization and control. Future work will be carried out to experimentally validate this framework in a controlled climate chamber environment, examining whether the noise of the mixed convection correction can be reduced and further assessing if globe thermometers are generally reliable instruments for *t*_*r*_ measurement.

## Methods

### Empirical model

To assess the basic contributions of forced and free convection about a black globe, Nusselt number correlations were obtained. The Nusselt number, *Nu*, is a dimensionless parameter that describes the ratio of convective heat transfer to conductive heat transfer, and is shown in Eq. 5. From *Nu*, convective heat transfer coefficients can be obtained as shown in Eq. (5), where *h*_*c*_ is the convective heat transfer coefficient [*Wm*^−2^ *K*^−1^], *D* is the hydraulic diameter [*m*], and *k* is the thermal conductivity of air [0.02662 *Wm*^−1^ *K*^−1^]. Therefore if *Nu* is known for a globe, the convective heat transfer coefficient can be calculated as in Eq. (6).

5 $$Nu=\frac{{h}_{c}D}{k}$$

6 $${h}_{c}=\frac{Nu\cdot k}{D}$$

Nusselt number correlation equations were obtained from the literature. Equation (7) shows the free convection correlation for free convection about a sphere^30^.

7 $$N{u}_{free}=2+\frac{0.589R{a}_{D}^{\frac{1}{4}}}{{(1+{(0.469/Pr)}^{\frac{9}{16}})}^{\frac{4}{9}}}$$

In eq. (7) *Ra* is the Rayleigh number, and *Pr* is the Prandtl number. Equation (7) is valid for *Ra* < 10^11^ and *Pr* ≥ 0.7. *Ra* is calculated in Eq. 8 and *Pr* is calculated in Eq. 9. In fact, it is this relationship to *D* hidden in *Ra* for free convection that is overlooked in the literature, and prevents direct comparison between Eq. 2 and other correlations proposed by Thorsson^10^, Humphreys^17^, and Fountain^13^.

8 $$Ra=\frac{g\beta }{\nu \alpha }({t}_{a}-{t}_{g}){D}^{3}$$

9 $$Pr=\frac{{c}_{p}\mu }{k}$$

In Eqs. 8 and 9, *g* is the acceleration due to gravity [9.81 *ms*^−2^], *β* is the thermal expansion coefficient of air [0.0034 *K*^−1^], *ν* is the kinematic viscosity [1.48  ⋅ 10^−5^ *m*^2^ *s*^−1^], *α* is the thermal diffusivity of air [2.591  ⋅ 10^−5^ *m*^2^ *s*^−1^], *c*_*p*_ is the specific heat capacity of air [1005 *Jkg*^−1^ *K*^−1^], and *μ* is the dynamic viscosity of air [1.81  ⋅ 10^−5^ *Pas*]. For comparison to the free convection case, the same forced convection relationships were derived using a Nusselt correlation provided in Eq. 10^31^.

10 $$N{u}_{forced}=2+(0.4R{e}^{\frac{1}{2}}+0.06R{e}^{\frac{2}{3}})P{r}^{0.4}$$

In Eq. 10, *Re* is the Reynolds number given in Eq. 11. Using these convection correlations, the magnitude of each mode of heat transfer was assessed for varying air velocities and globe temperatures. Equation 10 is valid for 3.5 < *Re* < 7.6 ⋅ 10^4^ and 0.7 < *Pr* < 380, appropriate for this application.

11 $$Re=\frac{{v}_{a}D}{\nu }$$

Often in convective processes, heat transfer is attributable to both forced and free convection, known as mixed convection. Often both contribute significantly, and ignoring one or simply summing heat transfer coefficients is not sufficient. Experimental data has shown that combining *Nu*_*free*_ and *Nu*_*forced*_ as in Eq. 12 with values of *n* = 3 or 4 being sufficient for most problems^32^.

12 $$N{u}_{mixed}={(N{u}_{free}^{n}+N{u}_{forced}^{n})}^{\frac{1}{n}}$$

To reparametrize a version of Eq. 1 for free, forced, or mixed convection effects on the *t*_*g*_ reading in environments with large surface to air temperature gradients, one can simply solve the energy balance between convection and radiation with *Nu* providing the convective heat transfer coefficient. The steady state equilibrium globe description equation, shown as Eq. 13 from^8^, will now becomes Eq. (14).

13 $$\sigma \epsilon ({t}_{g}^{4}-{t}_{r}^{4})={h}_{c}({t}_{a}-{t}_{g})$$

14 $${t}_{r}=\sqrt[4]{{t}_{g}^{4}+\frac{Nu\cdot k}{\epsilon \sigma D}({t}_{g}-{t}_{a})}$$

The form of Eq. 14 is useful since the correction can be applied for free, forced, and mixed convection attempting to obtain the best fit for experimental data.

### Experimental setup

Building off the previous simulation logic, it was desirable to conduct experiments to observe the error potentials realized in the built environment. To maximize the difference between the air temperature, *t*_*a*_, and the mean radiant temperature, *t*_*r*_, and therefore to radiatively force the globe temperature, *t*_*g*_, away from the air temperature, experiments were conducted in the Cold Tube radiant cooling pavilion.

**Figure 11:**
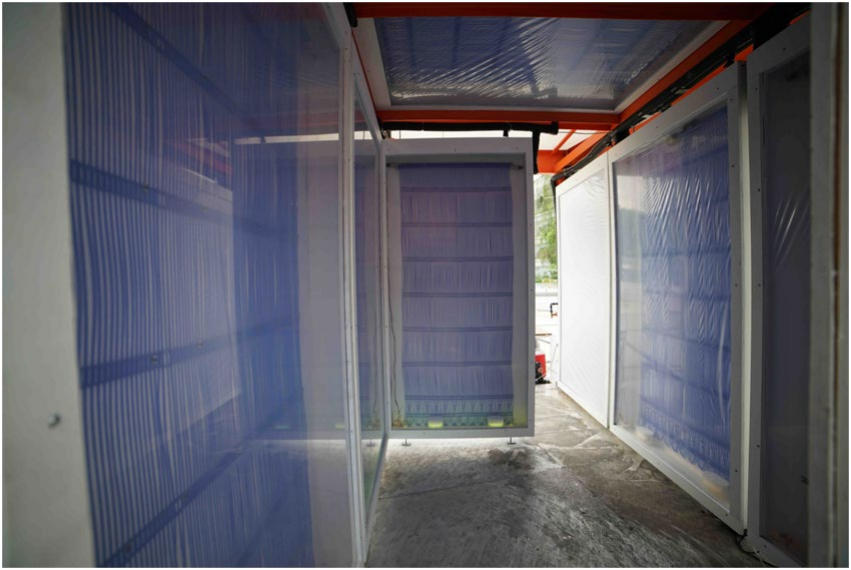
Membrane-assisted radiant cooling panels avoid both condensation and convection, the ideal environment for testing potential free convection error contributions.

The Cold Tube was a radiant cooling experimental pavilion outdoors in Singapore, demonstrating how comfort can be achieved with radiation, and not providing any air conditioning. While the primary technical mechanism of the Cold Tube pavilion was an infrared-transparent membrane to avoid condensation on the radiant cooling panels supplied with fluid below the dew point outdoors in humid environments, this mechanism also served to provide convective isolation. Since the cold surface was convectively isolated from the air in the space, the air temperature often did not drop more than 2 °C below the ambient condition. This feature made the Cold Tube a prime environment to test free convection affects on globe temperature readings, as it was designed to achieve up to 10 °C separation between *t*_*r*_ and *t*_*a*_. A photo of the Cold Tube is shown in Fig. 11.

Chilled water is supplied to the blue capillary mats in the panels in Fig. 11, at temperatures between 3 °C and *t*_*a*_. The pavilion was outside in Singapore, where the dewpoint consistently remained between 23 and 24 °C. The clear membrane was transparent to 80% of thermal radiation, characterized as in Teitelbaum *et al*.^33^.

The membrane was fixed to the panel, sealing the cold capillaries from the warm and humid air. Thermal radiation could travel through the membrane without cooling it, such that there was no mechanism to cool the air. Conduction and limited convection occurred, but at low rates compared to the radiation. The Cold Tube was comprised of 10 radiant cooling panels, 8 vertical panels and 2 horizontal across the top.

**Figure 12:**
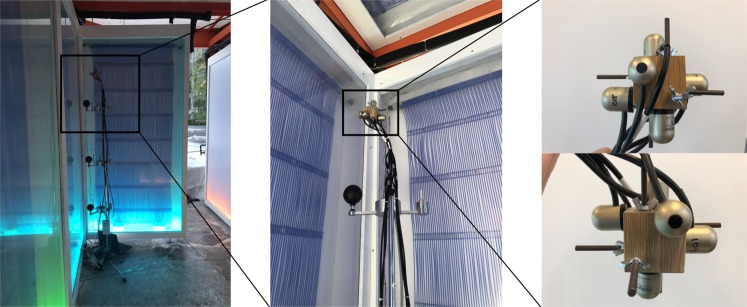
Measuring the mean radiant temperature in the Cold Tube with 6 pyrgeometers arranged orthogonally on a wooden cube alongside 4 black globes with anemometeors, air temperature and humidity sensors. The data from the top globe was used for this paper, as it was physically closest to the pyrgeometer.

**Figure 13:**
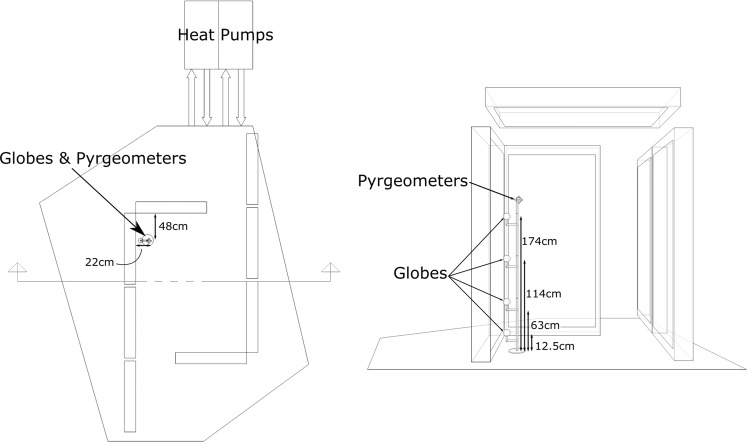
Section and plan diagrams of the Cold Tube showing radiant cooling panels and the position of the instrumentation rig.

Four globe measurement systems located at heights of 12.5, 63, 114, and 174 cm were placed in one corner of the Cold Tube as shown in photos in Fig. 12, and diagrammed with dimensions in Fig. 13. The globes were 100 mm in diameter. Each black globe was accompanied with an air temperature sensor, relative humidity sensor, and air speed sensor, comprising the ThermCondSys 5500 measurement system. The globe temperature and air temperature sensors are Pt-100 thermistors (±0.1 °C). The air temperature sensor was shielded from radiation with a highly reflective silver cone. The air speed sensor is a spherical omnidirectional air speed sensor and temperature compensation sensor, vacuum covered with an aluminum coating that increases their resistance to contamination and decreases the effect of thermal radiation on the accuracy of the measurement ( ±  0.02 *ms*^−1^). The relative humidity sensor has a  ± 2% accuracy. For the ground truth *t*_*r*_ measurements, a set of 6 radiometers (Apogee, SL-510-SS; 0.12 mV per *Wm*^−2^; 1% measurement repeatability; 5% calibration uncertainty;  ± °C) oriented orthogonally measuring radiant flux in all 6 cardinal directions. The cubical set of 6 radiometers was considered the ground truth for the measurement of *t*_*r*_ since the sensing element is not sensitive to convection. These 6 values were averaged and comprise the *t*_*r*_ at the measurement location. All sensors were calibrated within 1 month of the experiments. The 6 pyrgeometers were arranged on a small wooden cube (side length = 4.5 cm) so as to minimize offsets that would displace the sensors far from the point of measurement as in the rig proposed by Thorsson^10^. In small spaces, these offsets could skew readings significantly if held closer to cold sources at the reaches of the arms.

A pyrgeometer is a reasonable choice of ground truth instrument because of its measurement method. The pyrgeometer has embedded a high accuracy thermistor which continuously measures the device temperature, *t*_*pyrg*_. The pyrgeometer itself has a proportional voltage output, which corresponds to a radiant flux between the device and the surface in a 150° field of view. Knowing both the device temperature and the radiant flux, *Q*_*rad*_ in *Wm*^−2^ allows Eq. 15 to be solved for the average surface temperature, *t*_*avg*_. This reading is not sensitive to convection, has a settling time of less than one second, and when all 6 cardinal directions are measured produces an accurate value of *t*_*r*_ at the point of measurement.

15 $${Q}_{rad}=\sigma ({t}_{pyrg}^{4}-{t}_{avg}^{4})$$

Measurements were recorded for all devices at 2 second intervals, and 10 second and 5 minute wind speed averages were used to calculate *t*_*r*_ using Eqs. 1 and 2. 5 minute time averages are recommended for using eqs. 1 and 2^7^. The calculated mean radiant temperatures were compared with the measured mean radiant temperature from the pyrgeometers.

The pyrgeometers measure radiant flux in a 150 ° field of view as well as the temperature of the device, allowing accurate back-calculation of the average surfaces in the field of view. Averaging all 6 cardinal directions helps reduce the dependence on angle of incidence of radiation between surfaces and the pyrgeometer detector and eliminates occlusion. A pyranometer measuring shortwave radiation was also installed in the Cold Tube. The pavilion was shaded both with an opaque canvas fabric, as well as the steel and panel construction so there was no direct sky exposure. Shortwave radiation was measured at the site of the pyrgeometer sensor with a pyranometer (SP-510; 0.057 mV per *Wm*^−2^; 1% measurement repeatability), manually directed in all 6 cardinal directions. The incoming shortwave radiation averaged 6.8 ± 1.1 *Wm*^−2^ and was directionally invariant. The shortwave radiation in *Wm*^−2^ was added to the averaged pyrgeometer reading also in *Wm*^−2^ before being converted to a value for *t*_*r*_ using Eq. 16 and *S*_*tot*_ being the cumulative short- and averaged long-wave radiation measurement in *Wm*^−2^.

16 $${t}_{r}=\sqrt[4]{\frac{{S}_{tot}}{\sigma }}-273.15$$

Differences between the standard method of correcting *t*_*g*_ for convection to yield *t*_*r*_ as shown in eqs. 1 and 2 and the ground truth collected with the pyrgeometer can be compared to demonstrate the difference in radiant heat transfer, *ΔQ*_*rad*_ [*Wm*^−2^], an occupant would experience within each environment.

17 $$\Delta {Q}_{rad}=\sigma (({t}_{human}^{4}-{t}_{r,groundtruth}^{4})-({t}_{human}^{4}-{t}_{r,standard}^{4}))=\varepsilon \sigma ({t}_{r,standard}^{4}-{t}_{r,groundtruth}^{4})$$

According to the Zero Principle, "a thermometer can only measure its own temperature”. Fundamentally, the Pt-100 temperature sensor is measuring the air temperature inside the black globe, which is drawn towards the mean radiant temperature through radiation. This is the principle underlying black globe mean radiant temperature corrections. In contrast, the pyrgeometers are black body heat flux sensors, which through a back-calculation (shown in equation15) gives the black body temperature of the surroundings at the point of measurement. This procedure is outlined in standards^12^, and the resulting temperature reading will be referred to as the "ground truth” in this paper as it is the desired value to be extracted from the black globe measurement after an appropriate correction.

## References

[1] Carrier WH (1911). Rational psychometric formulae. Trans. Am. Soc. Mech. Eng..

[2] Fanger, P. O.* Thermal comfort. Analysis and applications in environmental engineering*. (Copenhagen: Danish Technical Press., 1970).

[3] Yagoglou C (1924). Report of committee to consider the report of the new york state commission on ventilation. Am. Soc. Heat.Vent. Eng..

[4] McIntyre, D. A. & Griffiths, I.Radiant temperature and thermal comfort. vol. CIB Commission W45 (1972).

[5] Singapore, S. Singapore standard ss 554: 2009: Code of practice for indoor air quality for air-conditioned buildings. *Building and Construction Standards Committe* (2009).

[6] Olesen BW (2012). Revision of en 15251: indoor environmental criteria. REHVA J..

[7] Standard 55, A. Thermal environmental conditions for human occupancy. *Am. Soc. Heating, Refrig. Air conditioning Eng*. (2017).

[8] De Dear R (1987). Ping-pong globe thermometers for mean radiant temperatures. Heat. Vent. Eng. J. Air Cond..

[9] Bedford T, Warner C (1934). The globe thermometer in studies of heating and ventilation. The J. Hyg..

[10] Thorsson S, Lindberg F, Eliasson I, Holmer B (2007). Different methods for estimating the mean radiant temperature in an outdoor urban setting. Int. journal climatology.

[11] Guo H, Teitelbaum E, Houchois N, Bozlar M, Meggers F (2018). Revisiting the use of globe thermometers to estimate radiant temperature in studies of heating and ventilation. Energy Build..

[12] ISO, I. 7726, ergonomics of the thermal environment, instruments for measuring physical quantities. *Geneva: Int. Standard Organ*. (1998).

[13] Fountain, W. H. Instrumentation for thermal comfort measurements: The globe thermometer. *Intern. study, Cent. for Built Environ*. (1987).

[14] Hey E (1968). Small globe thermometers. J. Phys. E: Sci. Instruments.

[15] Aitken J (1888). Addition to Thermometer Screens. Part IV. Proc. Royal Soc. Edinb..

[16] Middel A, Selover N, Hagen B, Chhetri N (2016). Impact of shade on outdoor thermal comfort: A seasonal field study in tempe, arizona. Int. journal biometeorology.

[17] Humphreys M (1977). The optimum diameter for a globe thermometer for use indoors. Annals Occup. Hyg..

[18] Graves K (1974). Globe thermometer evaluation. Am. Ind. Hyg. Assoc. J..

[19] Hill L, Vernon HM, Hargood-Ash D (1922). The kata-thermometer as a measure of ventilation. Proc. Royal Soc. Lond. B: Biol. Sci..

[20] HILL LEONARD (1920). THE SCIENCE OF VENTILATION AND OPEN-AIR TREATMENT. Monthly Weather Review.

[21] Armspach O, Ingels M (1922). Temperature, humidity, and air motion effects in ventillation. Am. Soc. Heat. Vent. Eng..

[22] Vernon H, Warner C (1932). The influence of the humidity of the air on capacity for work at high temperatures. J. Ind. Hyg..

[23] Yagoglou C (1926). Effective temperatures versus kata-thermometer: A reply to h.m. vernon. J. Ind. Hyg..

[24] Marino, C., Nucara, A., Pietrafesa, M., Polimeni, E. & Costanzo, S. Outdoor mean radiant temperature estimation: Is the black-globe thermometer method a feasible course of action? In *2018 IEEE International Conference on Environment and Electrical Engineering and 2018 IEEE Industrial and Commercial Power Systems Europe (EEEIC/I & CPS Europe)*, 1–7 (IEEE, 2018).

[25] Ličina VF (2018). Development of the ashrae global thermal comfort database ii. Build. Environ..

[26] Raftery, P., Duarte, C., Schiavon, S. & Bauman, F. A new control strategy for high thermal mass radiant systems (2017).

[27] Chinazzo G, Wienold J, Andersen M (2019). Daylight affects human thermal perception. Sci. reports.

[28] Hoyt T, Arens E, Zhang H (2015). Extending air temperature setpoints: Simulated energy savings and design considerations for new and retrofit buildings. Build. Environ..

[29] Teitelbaum E, Jayathissa P, Miller C, Meggers F (2020). Design with comfort: Expanding the psychrometric chart with radiation and convection dimensions. Energy Build..

[30] Churchill S, Bernstein M (1977). A correlating equation for forced convection from gases and liquids to a circular cylinder in crossflow. J. Heat Transf..

[31] Whitaker S (1972). Forced convection heat transfer correlations for flow in pipes, past flat plates, single cylinders, single spheres, and for flow in packed beds and tube bundles. AIChE J..

[32] Siebers D.*Experimental mixed convection heat transfer from a large, vertical surface in a horizontal flow*. Ph.D. thesis, Stanford University, Palo Alto, CA (1983).

[33] Teitelbaum E (2019). Revisiting radiant cooling: condensation-free heat rejection using infrared-transparent enclosures of chilled panels. Archit. Sci. Rev..

